# Insulin Treatment Attenuates Decline of Muscle Mass in Japanese Patients with Type 2 Diabetes

**DOI:** 10.1007/s00223-017-0251-x

**Published:** 2017-02-28

**Authors:** Ryotaro Bouchi, Tatsuya Fukuda, Takato Takeuchi, Yujiro Nakano, Masanori Murakami, Isao Minami, Hajime Izumiyama, Koshi Hashimoto, Takanobu Yoshimoto, Yoshihiro Ogawa

**Affiliations:** 1 Department of Molecular Endocrinology and Metabolism, Graduate School of Medical and Dental Sciences, Tokyo Medical and Dental University, 1-5-45 Yushima, Bunkyo-ku, Tokyo, 113-8519 Japan; 2 Center for Medical Welfare and Liaison Services, Tokyo Medical and Dental University, Tokyo, Japan; 3 Department of Preemptive Medicine and Metabolism, Graduate School of Medical and Dental Sciences, Tokyo Medical and Dental University, Tokyo, Japan; 4 CREST, Japan Agency for Medical Research and Development, Tokyo, Japan

**Keywords:** Sarcopenia, Insulin, Type 2 diabetes, Diabetes treatment

## Abstract

Sarcopenia is defined as an age-related loss of skeletal muscle mass and strength, and is a major cause of disability and mobility limitations. Recent studies have demonstrated that type 2 diabetes and insulin signaling deficiencies contribute to the progression of sarcopenia, suggesting that a sufficient supply of insulin to the skeletal muscles may be important for the maintenance of muscle function; however, little has been reported regarding whether insulin treatment can protect against sarcopenia. We conducted a retrospective observational study to examine the impact of insulin treatment on the muscle mass of patients with type 2 diabetes. A total of 312 patients (mean age: 64 ± 11 years; 40.8% female; 27.6% treated with insulin) were studied in this retrospective observational study. Skeletal muscle index (SMI) and grip strength (kg) were used to assess sarcopenia. The prevalence of sarcopenia was 18.0%. Insulin treatment was shown to be protective against the annual decline of SMI (standardized β 0.195; p = 0.025) even after adjusting for covariates, including age, gender, duration of diabetes, and body mass index. In a cohort matched by propensity scores, insulin treatment significantly increased the 1-year change in SMI (mean ± SE) compared with non-insulin-treated group (2.40 ± 0.98% vs. −0.43 ± 0.98%; p = 0.050). Our data suggest that insulin treatment could attenuate the progression of sarcopenia in patients with type 2 diabetes.

## Introduction

Sarcopenia is defined as the loss of skeletal muscle mass and strength that occurs with aging and is a major cause of disability and mobility limitations [1–3]. Although it has been clearly shown that sarcopenia has an adverse effect on mobility, the quality of life (QOL), and mortality [2, 4], the diagnostic thresholds of muscle mass and strength remain controversial. In 2010, the European Working Group on Sarcopenia in Older People was the first to achieve a consensus on the definition of sarcopenia, including the diagnostic thresholds for muscle mass and strength [1]. Subsequently, similar definitions were created by the International Working Group on Sarcopenia [2] and Asian Working Group for Sarcopenia (the criteria for Asian population) [3]. However, these definitions were determined based on the results of various studies using individual definitions of sarcopenia and diagnostic thresholds in each study. In addition, it remains uncertain whether the consensus made much such groups is applicable to patients of any race or those with chronic diseases. Of the chronic diseases, diabetes has been reported to be one of the significant contributors to the exacerbation of sarcopenia [5–8]. The Health, Aging, and Body Composition Study revealed that elderly patients with diabetes have lower muscle strength than non-diabetic subjects [6]. Using a longitudinal survey, this research group also reported that patients with diabetes have an increased risk for the excessive loss of both skeletal muscle mass and strength, particularly in the lower extremities [7, 8]. Given these findings, patients with diabetes are thought to be susceptible to mobility-related disabilities through the loss of muscle mass and strength primarily in the lower extremities.

Resistance training with protein supplementation was reported to be the most effective form of treatment for sarcopenia, [9, 10]. Pharmaceutical therapies have also been studied; however, there is insufficient evidence supporting effective medication for the treatment of sarcopenia [11]. In diabetic patients, poor glycemic control and insulin resistance are independently associated with the decline of skeletal muscle mass among elderly patients with diabetes [12, 13]. Consequently, these abnormalities could be considered a therapeutic target for sarcopenia. Indeed, it was reported that insulin sensitizers may attenuate the decline in muscle mass in patients with diabetes [14]. In addition, low endogenous insulin secretion was also associated with muscle mass among patients with diabetes [15]. Furthermore, supraphysiological hyperinsulinemia has been reported to be necessary for the stimulation of muscle protein synthesis and anabolic signaling in elderly subjects [16], suggesting that sufficient insulin supply could provide protection against the progression of sarcopenia in patients with diabetes. Therefore, we investigated the impact of insulin treatment on sarcopenia in patients with type 2 diabetes.

## Methods

### Subjects

**Fig. 1:**
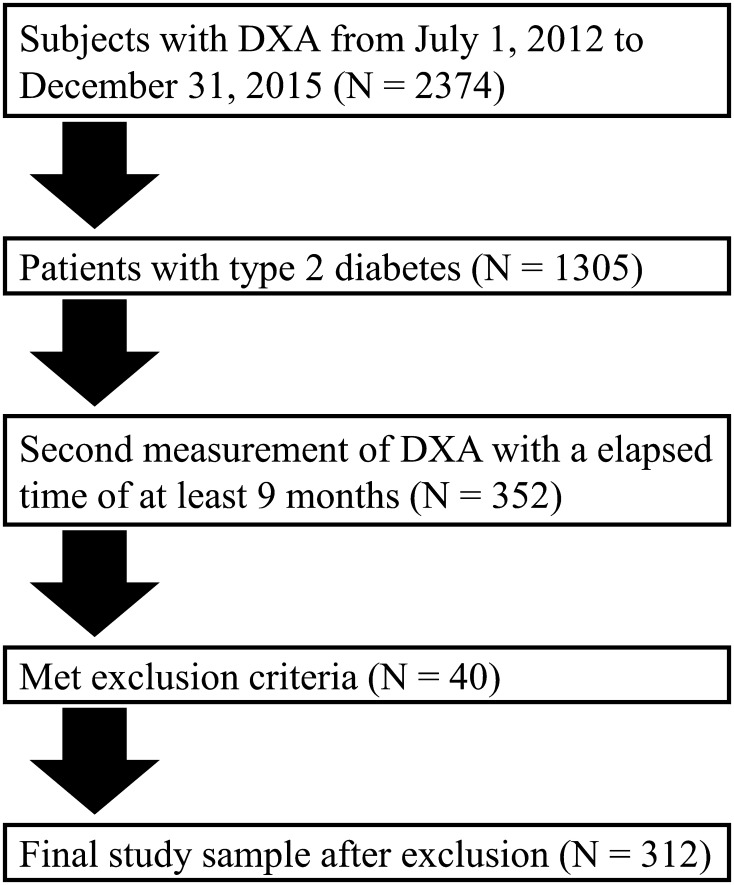
Flow chart

**Fig. 2:**
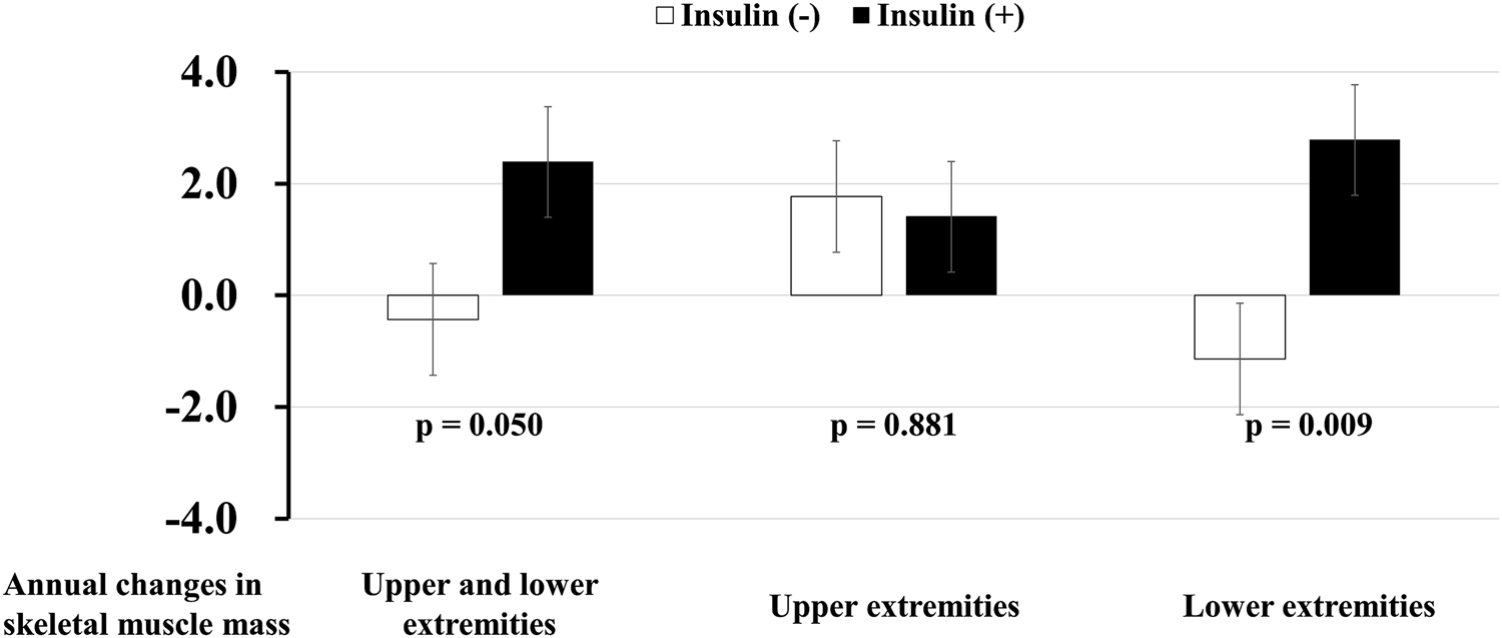
Annual changes in skeletal muscle index (mean ± SE, %) in appendicular (*left*), upper extremities (*middle*), and lower extremities (*right*) in patients with type 2 diabetes in the propensity score-matched cohort. *White* and *black bars* indicate non-insulin-treated and insulin-treated group

We screened type 2 diabetic patients aged older than 20 years who regularly visited the Tokyo Medical and Dental University Hospital for at least 1 year and had undergone a whole-body dual-energy X-ray absorptiometry (DXA) from July 1, 2012 to December 31, 2015. As shown in Figs. 1, 2, 374 patients aged more than 20 years at our hospital had undergone a whole-body DXA during the study period, of whom 1305 patients were diagnosed as having type 2 diabetes. We selected 352 patients with type 2 diabetes who had undergone a second DXA measurement with an elapsed time greater than 9 months to evaluate body composition. Exclusion criteria included severe liver disease, renal impairment (estimated glomerular filtration rate (GFR) [eGFR] < 15 mL/min/1.73 m^2^ or undergoing renal replacement therapy), pregnancy, infectious diseases, and cancer. Finally, 312 patients were enrolled in this retrospective study (Fig. 1). The median with interquartile range of the period between the first and second DXA measurement was 1.04 years (0.94–1.42 years). The patients were divided into an insulin-treated group (N = 86) and non-insulin-treated group (N = 216). The present study complied with the Declaration of Helsinki and was approved by the research ethics committee of Tokyo Medical and Dental University.

### Measurement of Body Composition

To evaluate the SMI, fat and fat-free tissue mass were measured using whole-body DXA (Lunar iDXA, GE Healthcare, Madison, WI). The patients were positioned for whole-body scans in accordance with the manufacturer’s protocol. The whole-body fat-free mass was divided into several regions (e.g., arms, legs, and trunk). Appendicular muscle mass was estimated as the sum of the fat-free mass of both the upper and lower limbs. The SMI was calculated as the appendicular muscle mass divided by the square of the height (kg/m^2^). Grip strength (kg) was measured using the hand dynamometer Grip-D (TKK5401, Takei, Niigata, Japan). The average grip strength was used for muscle strength. According to the Asian sarcopenia criteria [3], an SMI <7.0 in males and <5.4 in females, as well as a grip strength <26.0 kg in males and <18.0 kg in females were defined as low muscle mass and reduced grip strength, respectively. Eventually, the patients with both low muscle mass and reduced grip strength were diagnosed with sarcopenia. The annual changes in SMI, and the muscle mass in the upper and lower extremities (%/year) were also determined.

### Clinical and Biochemical Analysis

Information was obtained from the patients’ medical records regarding the medications for diabetes, dyslipidemia, hypertension, hyperuricemia, and anti-platelet agents, duration of diabetes, diabetic retinopathy, history of cardiovascular disease (CVD), and smoking status. CVD was defined as the presence of a previous stroke, myocardial infarction, or coronary revascularization procedure. Smoking history was classified as either current or non-smokers. The systolic and diastolic blood pressures (SBP and DBP) were measured in a sitting position after at least 5 min rest, using an electronic sphygmomanometer (ES-H55, Terumo Inc., Tokyo, Japan). The BMI was calculated as the weight divided by the square of the height (kg/m^2^). Routine tests included alanine transaminase (ALT), aspartate transaminase (AST), gamma-glutamyl transpeptidase (γ-GTP), high-density lipoprotein (HDL) and low-density lipoprotein (LDL) cholesterol, triglycerides, and uric acid. All tests were determined using standard laboratory procedures. The triglycerides-to-HDL cholesterol (TG/HDL-C) ratio was used for the assessment of insulin resistance. HbA1c was measured using the latex agglutination method. Urinary albumin and creatinine excretion were measured in a spot urine collection by a turbidimetric immunoassay and enzymatic method. The ratio (ACR, mg/g) was used for the assessment of albuminuria. The GFR, ml/min/1.73 m^2^ was calculated using the equation for Japanese [17].

### Statistical Analysis

Statistical analysis was carried out using IBM SPSS version 21.0 (IBM, Armonk, NY, USA), and the results were expressed as the mean ± SD, median, and interquartile range or percentages. A T-test, Mann–Whitney U test, or Chi-square test as appropriate were used for group comparisons (with insulin vs. without insulin). Linear regression analyses with a stepwise procedure were used to assess the factors associated with a 1-year change in SMI. The examined putative risk factors consisted of the duration of diabetes, smoking status, history of CVD, grip strength, BMI, SBP, TG/HDL-C ratio, HbA1c, eGFR, as well as the use of insulin, oral hypoglycemic agents, angiotensin receptor blockers, and statins (multivariate model 1). Age and gender were forced into the models because these are strong determinants of muscle mass and function. We also determined the 1-year changes in HbA1c, BMI, and other markers for cardiometabolic risks, including lipid and uric acid levels. We also examined the correlation for the change in SMI with the changes in these parameters (Pearson correlation). We then created a multivariate regression model (multivariate model 2) in which the covariates that were significantly correlated with a change in SMI and the covariates selected in the multivariate model 1 were entered. Finally, we performed propensity score (PS) matching to eliminate any possible treatment bias. The PS was calculated using multivariable logistic regression models that included the following parameters: age, gender, BMI, HbA1c, and duration of diabetes, logarithmically transformed urinary ACR, and eGFR. For the calculation of the PS, the dependent variable was insulin treatment at baseline. We performed 1:1 matching on the PS using nearest neighbor matching with a maximum caliber of 0.01 of the PS. The annual change in SMI (mean ± SE) was compared between the insulin-treated and non-insulin-treated patients using analysis of covariance (ANCOVA). The differences were considered to be statistically significant at a p-value less than 0.05.

## Results

A total of 312 Japanese patients with type 2 diabetes (mean age: 64 ± 11 years; 40.8% female; range: 21–89 years old) were studied. Table 1 presents the clinical characteristics of the study participants. The insulin-treated patients exhibited significantly higher levels of SBP and urinary ACR, a longer duration of diabetes, higher prevalence of diabetic retinopathy, and lower levels of grip strength, triglycerides, AST, ALT, and eGFR than the non-insulin-treated patients. As shown in Table 2, the insulin-treated patients were significantly more likely to receive diuretics and calcium channel blockers, but less likely to receive sulfonylureas and dipeptidyl peptidase-4 (DPP4) inhibitors than the non-insulin-treated patients.

**Table 1:** Clinical characteristics in patients with type 2 diabetes

	Insulin (−) (N = 216)	Insulin (+) (N = 86)	p-values
Age (years)	63 ± 12	66 ± 12	0.031
Gender (% female)	38	42	0.610
Systolic blood pressure (mmHg)	126 ± 15	130 ± 16	0.031
Diastolic blood pressure (mmHg)	75 ± 13	75 ± 15	0.774
Body mass index (kg/m^2^)	25.1 ± 4.6	24.3 ± 3.8	0.172
Grip strength (kg)	29.4 (20.3–36.5)	22.9 (17.2–30.3)	0.001
Skeletal muscle index	6.7 (5.9–7.6)	6.4 (5.8–7.2)	0.263
Body fat (%)	33.6 ± 7.1	34.3 ± 8.5	0.549
Duration of diabetes (years)	6 (3–9)	10 (3–18)	<0.001
Proliferative diabetic retinopathy (%)	2	17	<0.001
Previous cardiovascular disease (%)	11	9	0.729
Current smoking (%)	11	13	0.501
HbA1c (mmol/mol)	51.7 ± 15.4	61.9 ± 15.4	<0.001
HbA1c (%)	6.9 ± 1.4	7.8 ± 1.4	
TG (mmol/l)	1.37 (1.02–2.35)	1.15 (0.89–2.02)	0.034
HDL cholesterol (mmol/l)	1.49 ± 0.42	1.58 ± 0.45	0.133
TG/HDL cholesterol ratio	2.15 (1.39–3.95)	1.91 (1.13–3.78)	0.271
LDL cholesterol (mmol/l)	2.82 (2.41–3.34)	2.84 (2.22–3.49)	0.416
AST (U/l)	23 (21–32)	21 (19–25)	0.004
ALT(U/l)	23 (17–35)	18 (15–27)	0.001
γ-GTP (U/l)	35 (22–62)	31 (20–46)	0.051
Uric acid (μmol/l)	312 (274–364)	312 (262–372)	0.648
Albumin-to-creatinine ratio (mg/g)	22 (14–59)	32 (14–133)	0.042
eGFR (ml/min/1.73 m^2^)	75.5 ± 19.7	69.6 ± 27.6	0.035

*ALT* alanine transaminase, *AST* aspartate transaminase, *eGFR* estimated glomerular filtration ratio, *GTP* glutamyl transpeptidase, *HDL* high-density lipoprotein, *LDL* low-density lipoprotein, *TG* triglycerides

**Table 2:** Medications for patients with type 2 diabetes

	Insulin (−) (N = 216)	Insulin (+) (N = 86)	p-values
Sulfonylureas (%)	18	4	0.002
Biguanides (%)	25	22	0.662
Alpha-GIs (%)	5	8	0.241
Glinides (%)	3	2	0.817
TZDs (%)	5	4	0.861
DPP4 inhibitors (%)	36	22	0.021
GLP1-R agonists (%)	2	1	0.666
SGLT2 inhibitors (%)	2	0	0.369
ACEIs (%)	4	3	0.775
ARBs (%)	33	41	0.153
Calcium channel blockers (%)	27	46	0.001
Beta-blockers (%)	12	9	0.553
Alpha-blockers (%)	2	2	0.799
Diuretics (%)	7	16	0.014
Statins (%)	29	39	0.076
Fibrates (%)	2	4	0.289
Ezetimibe (%)	5	2	0.280
Uric acid-lowering agents (%)	7	5	0.539
Anti-platelet agents (%)	11	18	0.116

*ACEIs* angiotensin converting enzyme inhibitors, *ARBs* angiotensin receptor blockers, *DPP4* dipeptidyl peptidase 4, *GIs* glycosidase inhibitors, *GLP1-R* glucagon-like peptide 1 receptors, *SGLT2* sodium–glucose cotransporter 2, *TZDs* thiazolidinediones

As shown in Table 3, insulin treatment was found to be protective against the annual decline of SMI in the univariate and gender- and age-adjusted models. In the multivariate model, which includes the duration of diabetes and BMI in addition to age and gender as covariates (multivariate model 1), the patients with treated with insulin were at a significantly lower risk for the decline of SMI than those who did not receive insulin treatment. In addition, insulin treatment was significantly and positively associated with the annual change in the muscle mass of the lower extremities (standardized β 0.213; p = 0.015) but not with that of the upper extremities (standardized β −0.012; p = 0.892) in the multivariate models. Since the 1-year change in SMI was significantly correlated with the BMI (r = 0.279; p < 0.001) and HbA1c (r = −0.162; p = 0.043) levels, we constructed a multivariate model in which the changes in BMI and HbA1c were added as covariates (multivariate model 2). In the model, insulin treatment was shown to be protective against a decline in SMI with a statistical significance. Insulin treatment was significantly associated with SMI in the lower but not the upper extremities in the multivariate models. Other significant covariates included in the model were the duration of diabetes and BMI at baseline. Although grip strength was strongly correlated with SMI (r = 0.637; p < 0.001) and SMI in the upper (r = 0.733; p < 0.001) and lower (r = 0.570; p < 0.001) extremities at baseline, grip strength was not correlated with the change in SMI (r = −0.032; p = 0.674) and SMI in the upper (r = 0.031; p = 0.680) and lower (r = −0.050, p = 0.509) extremities. Finally, grip strength was not selected in the multivariate models.

**Table 3:** Factors associated with 1-year change in skeletal muscle mass in patients with type 2 diabetes

	SMI		SMI in the upper extremities		SMI in the lower extremities	
	Standardized β	p-values	Standardized β	p-values	Standardized β	p-values
Univariate	(A-R^2^ = 0.114)		(A-R^2^ = 0.001)		(A-R^2^ = 0.119)	
Insulin	0.177	0.041	−0.023	0.760	0.169	0.045
Age- and gender-adjusted	(A-R^2^ = 0.117)		(A-R^2^ = 0.008)		(A-R^2^ = 0.123)	
Insulin	0.175	0.042	−0.012	0.873	0.174	0.038
Age	−0.064	0.471	0.074	0.328	−0.049	0.514
Gender	−0.021	0.983	0.054	0.468	−0.042	0.575
Multivariate 1	(A-R^2^ = 0.165)		(A-R^2^ = 0.008)		(A-R^2^ = 0.197)	
Insulin	0.195	0.025	−0.012	0.892	0.213	0.015
Age	−0.139	0.132	0.074	0.328	−0.071	0.387
Gender	0.024	0.771	0.054	0.468	−0.030	0.682
Duration of diabetes	−0.301	0.001	NS		−0.182	0.021
Body mass index	−0.188	0.036	NS		−0.220	0.005
Multivariate 2	(A-R^2^ = 0.177)		(A-R^2^ = 0.018)		(A-R^2^ = 0.215)	
Insulin	0.184	0.042	−0.023	0.768	0.173	0.035
Age	−0.062	0.554	−0.035	0.664	−0.024	0.782
Gender	0.007	0.933	0.061	0.427	−0.018	0.810
Duration of diabetes	−0.298	0.001	NS		−0.166	0.038
BMI	−0.208	0.042	NS		−0.180	0.027
Change in BMI	0.080	0.418	0.094	0.228	0.138	0.075
Change in HbA1c	−0.097	0.272	−0.054	0.497	−0.044	0.562

*A-R* ^*2*^ adjusted R^2^, *BMI* body mass index, *NS* not selected in the model, *SMI* skeletal muscle index

In a propensity-matched cohort (Fig. 2), insulin treatment significantly increased the SMI in 1-year (mean ± SE) compared with the non-insulin-treated group (2.40 ± 0.98% vs. −0.43 ± 0.98%; p = 0.050). When the effect of insulin was determined separately for the muscle mass of the upper and lower extremities, insulin significantly attenuated the decline of muscle mass in the lower extremities but not in the upper extremities (Fig. 2).

Finally, we investigated whether the change in SMI in patients treated with insulin could be correlated with body fat (android and gynoid) and cardiometabolic risk factors. The change in SMI was not correlated with that in the android (r = 0.092; p = 0.247) and gynoid (r = 0.031; p = 0.409). In addition, the change in SMI was significantly correlated with that in HbA1c (r = −0.150; p = 0.047), HDL cholesterol (r = 0.244; p = 0.034), and uric acid (r = −0.310; p = 0.009) but not with triglycerides (r = −0.068; p = 0.308), LDL cholesterol (r = −0.049; p = 0.360), AST (r = −0.010; p = 0.471), ALT (r = −0.013; p = 0.460), or gamma GTP (r = 0.092; p = 0.247).

## Discussion

Diabetes is a strong risk factor for the progression of sarcopenia and insulin activity is critical to maintain the balance of muscle protein synthesis and degradation [5]. There is a close association between insulin resistance and sarcopenia. Skeletal muscle is highly responsible for insulin-stimulated glucose uptake which accounts for approximately 75% of the glucose uptake of the entire body. It has been suggested that people with high muscle mass could have increased capacity of glucose uptake and insulin sensitivity in the skeletal muscle and vice versa. In observational and interventional studies [14, 18], insulin sensitizers can increase muscle mass with an improvement in mitochondrial activity and decreased protein degradation in skeletal muscle mass. However, it is uncertain whether insulin treatment could attenuate the loss of skeletal muscle mass and/or function [19, 20]. In addition, the long-term effects of insulin treatment on muscle mass and strength are unknown. In this study, we revealed that insulin treatment significantly attenuated the decline of skeletal muscle mass in patients with type 2 diabetes. This association remained when we used PS matching.

The protective effect of insulin on the reduction of muscle mass was primarily observed in the lower extremities (Table 3; Fig. 2). Although the protective mechanism of insulin on the progression of muscle dysfunction that was dominant in the lower extremities remains unclear, some previous reports provide important speculation [21, 22]. Patients with diabetes, especially those with a longer duration of the disease are at an increased risk for poor muscle strength in the quadriceps [21] and walking impairments [22]. These data suggest the possibility that patients with a long duration of diabetes are more likely to develop muscle dysfunction in the lower extremities rather than in the upper extremities. Compared with the diabetic patients who have had the disease for a short duration, those with a long duration exhibit low endogenous insulin levels in the bloodstream, implying that insulin signaling in the skeletal muscle may be impaired. This results in low muscle mass and decreased function in diabetic patients with a long duration of diabetes. Thus, it is possible that an efficient supply of exogenous insulin could improve insulin signaling in the skeletal muscle, promote protein synthesis, and protect against the loss of muscle mass among patients with diabetes, especially those with a long duration of diabetes. In addition, we used a multivariate model to identify the 1-year changes in the HbA1c and BMI levels as covariates that insulin treatment remained to significantly increase muscle mass. These findings provide evidence that the protective effects of insulin treatment on the decline of muscle mass may be independent of an improvement in glycemic control or load increase. Therefore, the resolution of the relative insufficiency of insulin via exogenous insulin injections may be the main factor with regards to the protection of muscle mass in this study. We further determined whether the change in SMI in patients with insulin treatment was correlated with that in body fat and cardiometabolic risk factors. We found that the change in SMI was indeed correlated with the change in HbA1c, HDL cholesterol, and uric acid. These data suggest that the increase in muscle mass induced by insulin treatment may reflect an improvement in peripheral insulin resistance. Based on our findings and the results from previous studies [21, 22], insulin treatment could be plausible means of preserving muscle function while also improving the glycemic control of patients with diabetes who are affected by low muscle mass and strength in the lower extremities.

Combined with the potential of insulin treatment to preserve muscle function and mass, it should be emphasized that insulin treatment should be initiated with the careful consideration of adverse effects, particularly hypoglycemia. Hypoglycemia increases the risk of dementia, falls, cardiovascular events and mortality in elderly patients with diabetes [23]. In particular, falls are the major cause of injury and a significant source of morbidity and disability which can severely reduce the QOL among the elderly. Comorbidities (e.g., chronic kidney disease) further increase the risk for hypoglycemia in elderly patients with diabetes. In addition, insulin treatment has the potential to increase body fat. The accumulation of body fat, particularly abdominal visceral fat, has been recognized as a strong contributor to the development of CVD and metabolic disorders, including diabetes [24, 25]. Therefore, physicians should balance the potential benefits and risks when recommending the use of insulin.

In addition to insulin treatment, the durations of diabetes and BMI were found to be significant predictors for the changes in SMI in this study (Table 3). Moreover, the presence of diabetes, poor glycemic control, and insulin resistance have been reported to increase the risk for lowering muscle mass and function in elderly individuals [5, 7, 8, 13, 26]. Therefore, it is conceivable that patients with a long duration of diabetes may be exposed to hyperglycemia and insulin resistance much longer than those with a short duration of diabetes, resulting in a reduction of muscle mass and function. In contrast, the negative association of the BMI with a change in SMI seems odd since patients with a high BMI are subjected to a high load (high body weight) and may be protective against the loss of muscle mass and function. One potential explanation is that patients with a high BMI are more likely to be obese and those who lost more weight (fat and non-fat mass) than non-obese patients via calorie restriction during the follow-up period, would exhibit a reduction in the SMI; however, no information regarding diet was available in this study.

This study has several limitations: (1) generalization of the findings of this study is limited due to the study design (hospital-based study including only Japanese individuals with type 2 diabetes); (2) propensity scores do not eliminate bias if there are unmeasured confounders; (3) information regarding changes in grip strength was not available and the follow-up period for muscle mass was relatively short; (4) we were unable to obtain data regarding diet and exercise, or diabetic neuropathy, all of which may affect muscle function; (5) we measured the grip strength in both the dominant and non-dominant hands once and used the average values to assess the degree of muscle strength in this study. Recently, a standardized protocol for grip strength measurements was suggested by Roberts HC et al. to improve the assessment of sarcopenia [27]. The protocol proposes that three measurements of grip strength should be conducted for each hand, and the maximal grip score obtained from all six trials should be used as the grip strength in the statistical analysis. Therefore, we should have measured a grip strength in accordance with this protocol; and (6) there was a significant difference in the age between the patients treated with insulin and those who were not. Sarcopenia occurs with advancing age (aging is the strongest contributor to the progression of sarcopenia); therefore, age differences could influence the outcome data, particularly regarding the effect of insulin treatment on muscle mass; however, insulin treatment was significantly associated with preserved muscle mass even after adjusting for covariates, including aging. Furthermore, it should be considered that the older age of patients receiving insulin treatment may underestimate but not overestimate the favorable effects of insulin treatment on muscle mass.

In summary, our data suggest that patients with type 2 diabetes treated with insulin may be at a lower risk for the loss of skeletal muscle mass in the lower extremities, compared with those who do not receive insulin treatment. Whether the administration of insulin influences the incident sarcopenia in randomized controlled trials remains to be elucidated.
